# Cobalt-Containing Dispersion Catalysts for Three-Phase Fischer–Tropsch Synthesis

**DOI:** 10.3389/fchem.2020.567848

**Published:** 2020-11-16

**Authors:** Anton Lvovich Maximov, Mayya Valerevna Kulikova, Oksana Sergeevna Dementyeva, Anna Konstantinovna Ponomareva

**Affiliations:** ^1^A. V. Topchiev Institute of Petrochemical Synthesis, Russian Academy of Sciences (RAS), Moscow, Russia; ^2^Faculty of Fundamental Physics and Chemical Engineering, Lomonosov Moscow State University, Moscow, Russia

**Keywords:** three-phase Fischer-Tropsch synthesis, cobalt catalysts, dispersed phase catalysis, nanoheterogeneous catalysis, slurry-reactor

## Abstract

Nanosized catalyst dispersions have significant potential for improving hydrocarbon production from carbon monoxide and hydrogen via Fischer–Tropsch synthesis, an essential alternative to the use of petroleum as a raw material. New dispersed cobalt catalysts and dispersed-phase cobalt-based catalysts with Pd, Al_2_O_3_, or ZrO_2_ additives for the Fischer–Tropsch synthesis were synthesized in the present work. A dispersed cobalt phase was prepared in a heavy paraffin medium using *ex situ* and *in situ* approaches through thermal decomposition of a nitrate precursor at various temperatures. Analyses showed that an increase in the temperature for catalytic suspension formation from 215 to 260°C enlarged the particles in the dispersed phase from 190 to 264 nm, which was probably due to increased agglomeration at elevated temperatures. The rheological properties of the obtained catalytic suspensions can be described by the Bingham equation. Furthermore, the concentration of the dispersed phase had a direct impact on the structure of the entire catalytic system. Ultrafine suspensions of palladium-promoted catalytic systems were tested for the Fischer–Tropsch synthesis. The overall yield of C_5+_ hydrocarbons was as high as 50 g/m^3^, and the productivity of the Pd-promoted catalytic systems reached 270–290 g/(kg_Co_ · h).

## Introduction

The production of liquid fuel is a priority for processing alternative raw materials (Dry, 2002; Neste Corporation, 2016; Douvartzides et al., 2019). The most unified technology for generating synthetic fuels is a two-stage method based on the partial oxidation of any carbon-containing feedstock to produce synthesis gas, or syngas (a mixture of carbon monoxide and hydrogen), followed by conversion to liquid hydrocarbons. The second stage of this process is referred to as the Fischer–Tropsch synthesis (FTS) (Mahmoudi et al., 2018), a catalytic process in which the catalyst precisely determines the yield and composition of the products and sets the requirements for the syngas properties. Thus, the FTS catalyst governs the concept of the entire method for producing synthetic petroleum products.

The synthesis of hydrocarbons from CO and H_2_ (FTS) is one of very few heterogeneous catalytic radical polymerization reactions (Henrici-Olivé and Olivé, 1984; Davis, 2001; Kollár et al., 2010). The FTS products are a mixture of hydrocarbons with a broad fractional composition from C_1_ to C_100_ and higher (Gorimbo et al., 2018). Group VIII bulk metals (mainly iron) or metal particles (iron or cobalt) supported on highly porous materials are commonly used as FTS catalysts (Van de Loosdrecht et al., 2013). The activity, stability, and selectivity of the catalytic system are largely determined by the shape and size of the particles of the active component (Khodakov et al., 2002). In particular, cobalt-containing catalysts enable the selective synthesis of liquid and solid straight-chain alkanes (Rafiq et al., 2011). It was previously established (Lapidus, 2013) that the active sites of cobalt FTS catalysts are bifunctional and composed of partially oxidized cobalt (in the +δ state) and cobalt oxide, which have Lewis acid properties and are responsible for partially drawing the electron density away from the metal. As-received cobalt-based catalysts consist of completely oxidized cobalt in the form of a salt or stable oxide distributed over the surface of a support, usually Al_2_O_3_ or SiO_2_ (Rathouský et al., 1991; Jacobs et al., 2002a, 2007). To activate the catalyst, the oxidized cobalt is reduced in hydrogen at a temperature above 350°C to form Co^0^. However, under these conditions, interactions between cobalt and the support often occur, resulting in hardly reduced compounds and thus a decrease in the reducibility of cobalt species (Jacobs et al., 2002b). Under the influence of a strong metal-support interaction, cobalt cannot be transformed into a null valence state. Co_3_O_4_ cobalt oxide, a support or mixed oxides, which result from the interaction of cobalt with a support during the pre-heating of the catalyst or its reduction, can play the role of a Lewis acid in the active site (Rytter et al., 2016).

Dispersed-phase catalysis, particularly chemical syntheses in the presence of a colloidal solution—an active phase of nanosized particles distributed in a dispersion medium—is a current new direction in catalysis (Khadzhiev, 2011). Colloidal dispersed-phase catalysts are characterized by a developed surface, and the absence of a carrier avoids the strong metal–carrier interactions that are typical of supported systems. These dispersions can be utilized in a slurry reactor, in which the nanoparticles of the active phase are dispersed in a liquid hydrocarbon medium (catalytic dispersion) (Khadzhiev et al., 2011). In free dispersion systems, there are no connections between the particles of the dispersed phase; thus, each particle is kinetically independent and, at sufficiently small sizes, participates in intense Brownian motion. Under isothermal conditions, coagulation (i.e., adhesion and the association of particles that retain their original shape and size, into dense aggregates may occur). The disperse composition of unstabilized dispersion systems continuously changes *via* particle enlargement up to complete segregation with the formation of separate macrophases. However, stabilized dispersions can remain unchanged for long time periods (Deryagin, 1979).

There are two ways to prepare a dispersed system: dispersion and condensation. Dispersion of macrophases resulting in the formation of lyophilic systems occurs spontaneously from the energy of thermal motion, which is enough for this process. On the other hand, the formation of lyophobic systems via the dispersion of a stable macrophase requires significant energy costs, which are determined by the total surface area of the dispersed-phase particles.

The condensation route for dispersion formation consists of the nucleation of a new phase or phases in a supersaturated metastable initial phase—the future dispersion medium. To prepare a finely dispersed system, there must be enough nuclei in the new phase, and their growth rate must not be too high. In addition, criteria for restricting excessive growth and adhesion of the dispersed-phase particles should be considered. The transformation of an initially stable homogeneous system into a metastable one can occur due to changes in the thermodynamic state parameters (pressure, temperature, composition). When the aggregative stability of the dispersion is impaired, particles associate irreversibly after approaching each other owing to Brownian motion, or the rate of aggregation becomes much higher than the rate of disaggregation (Efremov and Us'yarov, 1976; Derjaguin et al., 1987; Chen and Russel, 1991). As a result, point (atomic) contacts form between solid particles, which can then become phase (cohesive) contacts. For such systems, the loss of aggregate stability also means the loss of sedimentation stability.

Traditional catalytic dispersions used in three-phase FTS are known to tend toward sedimentation (Yurkov et al., 2007). Such suspensions containing particles 50–150 μm in size are thermodynamically unstable, and larger particles worsen the catalytic properties of the system significantly. As defined, nanoparticles have sizes ranging from 1 to 100 nm, although this range has been extended to the micron region. Particles of such dimensions can exhibit chemical and catalytic properties different from those of the bulk metal (Akcora et al., 2009; Hanemann and Szabó, 2010). To obtain nanoparticles, it is necessary to control not only the decomposition of metal salts but also to ensure sufficient stabilization of the product particles. Organic metal compounds, such as metal carbonyls, can be used as a precursor, and various organic compounds can be used as a solvent (stabilizer).

There are several techniques to prepare cobalt-containing catalytic dispersions for the three-phase FT process. For example, via the decomposition route, dispersions can be generated by injecting a toluene solution of cobalt carbonyl [Co_2_(CO)_8_] into molten trioctylphosphine oxide at 150°C (Puntes et al., 2001), or by injecting the same salt into *o*-dichlorobenzene into a boiling *o*-dichlorobenzene solution containing oleic acid and trioctylphosphine oxide (182–190°) (Puntes et al., 2002; Scariot et al., 2008). In one study, nanoparticles of metallic cobalt were obtained by the decomposition of cobalt carbonyl in a medium of ionic liquids at 150°C (Murray et al., 2000). In this case, spherical (~10 nm) and cubic (80–100 nm) nanoparticles were formed, the ratio between which was governed by the decomposition time.

The other approach to obtain cobalt dispersions involves the thermal decomposition of a precursor in preliminary formed microemulsions. Surfactants are often employed in this method; surfactant pairs such as oleic acid/oleylamine, oleic acid/trioctylphosphine oxide, and trioctylphosphine/oleylamine have been reported as suitable (Shao et al., 2006). For example, metallic cobalt nanoparticles 25 nm in size were synthesized from a solution of cobalt(II) acetate in trioctylamine in the presence of oleylamine, polyvinylpyrrolidone, and oleic acid under a nitrogen atmosphere at 260°C.

The literature data on three-phase FTS in the presence of nanoscale cobalt catalysts are scarce, although some works have been published. Kikuchi et al. studied the FT process in the presence of an ultrafine cobalt suspension with particle sizes of 7–9 nm under 20 atm and 210°C conditions (Kikuchi et al., 1999). The catalysts were prepared by the decomposition of cobalt carbonyl in ionic liquids. The conversion of CO reached 30%, and the TOF value (reaction rate) was 0.5 h^−1^. The main reaction products were C_7_-C_30_ hydrocarbons, including 79% paraffin. Nevertheless, it should be noted that exposing the catalyst to air resulted in its complete deactivation.

Recently, FTS was investigated in the presence of ultrafine cobalt catalysts prepared by chemical reduction in a liquid phase, where CoCl_2_ was reduced by KBH_4_ in a water-methanol solution (Gual et al., 2012). At 270° and 30 atm with a highly dilute catalyst (catalyst:liquid phase weight ratio of 1:400), the performance of the prepared catalytic system was 2–5-fold higher than that of the traditional Co–MgO–kieselguhr catalyst loaded in a fixed-bed reactor. The introduction of promoters, namely Mn, Ti, and Cr, reduced the catalyst particle size to 18–19 nm. Moreover, the conversion of CO reached 40–50%, and the α-value, which reflects the degree of polymerization according to the Anderson–Schulz–Flory model, was 0.82−0.84. When the particle size was reduced from 60 to 20 nm (i.e., a 3-fold decrease, the catalyst showed a 4-fold increase in productivity). However, with increasing cobalt catalyst nanoparticle size, the selectivity toward the target products (liquid hydrocarbons) decreased, whereas the selectivity toward gaseous hydrocarbons, in particular methane, increased remarkably.

Nanosized cobalt FTS catalysts have also been prepared by a method combining a sol-gel technique and drying in supercritical liquids (Li et al., 2009). Unlike conventional methods, drying solutions under supercritical conditions prevented the agglomeration of solid particles (Zhang et al., 2006) and afforded particle sizes of 10–15 nm. The complex 100Co:50Zn:10Mn:7K catalyst prepared by this approach in a three-phase system under supercritical conditions (280°C, 15 atm, catalyst:liquid phase weight ratio of 1:38) demonstrated a CO conversion of 92% and selectivity toward liquid products of 71%.

Interesting data were obtained in a study on FTS over *in situ* prepared ultrafine cobalt-containing catalysts (Kuz'min et al., 2018). The nanosized unpromoted cobalt catalyst was synthesized in a stirred reactor in the temperature range of 280–290° by *in situ* thermal decomposition of an aqueous solution of cobalt nitrate introduced into a liquid alkane solution of molten paraffin. In addition to the FT reaction, intense hydrogenolysis of the alkanes in the dispersion medium also occurred in the presence of the catalyst. As a result, a significant difference in the molecular weight distribution of the products was observed compared with that in the Anderson–Schulz–Flory model.

The aims of the present work were to study the formation of cobalt–hydrocarbon dispersion systems with a mixture of hydrocarbons as the dispersion medium; to investigate the catalytic properties of these dispersions for the FTS; and to determine the effect of the preparation conditions on the characteristics of the synthesized dispersions.

## Experiment

### Catalyst Preparation

Cobalt-containing dispersion catalysts for the FTS were prepared by thermal decomposition (thermolysis) of cobalt nitrate (Co(NO_3_)_2_·6H_2_O). Paraffin P-2 (RF state standard GOST 23683-89), a mixture of C_19_-C_35_ solid paraffin, was used as the dispersion medium. The amount of cobalt precursor was set to obtain 2–16 g of metallic Co (cobalt in a zero-valent state) in 100 ml of the dispersion medium. Three protocols with different methods for metal precursor introduction were applied for catalyst synthesis.

#### Protocol 1 (Fast Introduction)

The solid metal precursor (9.8 g) was added to 100 ml of the molten dispersion medium under stirring and argon flow (6 ml/h, 60 rpm). The temperature of the synthesis varied from 215 to 260°C. This range exceeds the cobalt nitrate decomposition temperature.

#### Protocol 2 (Slow Introduction)

The metal precursor salt was preliminarily dissolved in distilled water, and the aqueous solution was introduced dropwise into the stirred dispersion medium under the above reaction conditions.

#### Protocol 3 (*in situ* Injection)

The precursor salt solution was injected directly into the FTS reactor in which the dispersion medium was already loaded under H_2_ flow (10 l/h) at 20 atm and 300°C. The formed dispersion system was maintained under the same conditions for another 20 min.

Using protocol 2, a series of dispersed-phase cobalt-based catalysts with Al_2_O_3_ or ZrO_2_ additives were also synthesized using aluminum [Al(NO_3_)_3_·9H_2_O] and zirconium [Zr(NO_3_)_2_·H_2_O] nitrate, respectively, as additive precursors. Therefore, before introduction into the dispersion medium, a mixed aqueous solution of cobalt and the aluminum or zirconium precursor was prepared. In this case, the concentration of cobalt nitrate in the aqueous solution was kept constant to provide the desired amount of metallic Co in the final dispersion, while the concentration of the aluminum or zirconium nitrate varied. Following protocol 3, dispersed-phase cobalt-based catalysts promoted with palladium and alumina were also prepared using palladium chloride (PdCl_2_) and Al(NO_3_)_3_·9H_2_O, respectively, as precursors for the syntheses.

### Catalyst Characterization

#### Dynamic Light Scattering (DLS)

The particle sizes of the dispersed phase of the prepared cobalt-containing systems were determined by DLS on a Malvern Zetasizer Nano ZS instrument. Prior to analysis, 0.01 g of each cobalt-containing dispersion was dissolved in 10 ml of *n*-hexane with the addition of 5 wt.% surfactant (sodium dioctyl sulfosuccinate).

#### Rheological Studies

Rheological studies of the cobalt-containing dispersions were performed on a Physica MCR301 rheometer (Anton Paar, Germany). The tests were carried out in a cone–plane type measurement cell (diameter of 50 mm, cone angle of 1°) at 80°C. The measurement sensitivity was 0.003 Pa.

#### X-Ray Diffraction (XRD)

XRD patterns were recorded on a Rigaku Rotaflex D/max-RC instrument using CuKα radiation (λ = 0.154 nm) within the range of 2θ = 3°-50° with a step size of 0.04° and scanning speed of 4 min^−1^. The degree of crystallinity was calculated from the ratio of the peak areas (integrated intensities) of the crystalline and amorphous phases.

#### Transmission Electron Microscopy (TEM)

TEM was performed on a JEOL JEM-2100 instrument at an accelerating voltage of 200 kV. Before analyses, 0.05 g of each cobalt-containing dispersion sample was dissolved in 10 ml of cyclohexane and then placed on a copper grid coated with a formvar film. The particle size distribution was determined using the Micro-Manager software; no less than three view field locations, in which 80–100 particles were observed, were analyzed for each measurement.

### Catalytic Activity

The catalytic experiments were conducted in a 100-ml three-phase slurry reactor. Before testing, the as-prepared catalyst dispersions were activated under hydrogen flow (2 MPa) for 12 h at 300°C. The FTS was carried out at a feed (H_2_:CO = 1:2 mol/mol) flow rate of 20 l/h at 20 atm and temperatures increasing from 200 to 260° in steps of 10°C every 12 h. Gas and liquid sampling were performed every 8–12 h after each temperature increase. The inlet syngas and the reaction products were analyzed by gas-liquid chromatography as described in a previous paper (Kuz'min et al., 2019).

To evaluate the catalyst performance for the FTS, the following parameters were calculated: CO conversion (*X*_co_, wt.%; ratio of CO reacted to CO introduced); product yield (*Y*_*N*_, g/m^3^; amount of product *N* obtained after feeding of 1 m^3^ of syngas at STP); and product selectivity (*S*_*N*_, %; ratio of carbon used in product *N* formation to total carbon supplied).

## Results

### Formation of Cobalt–Paraffin Dispersions

**Figure 1:**
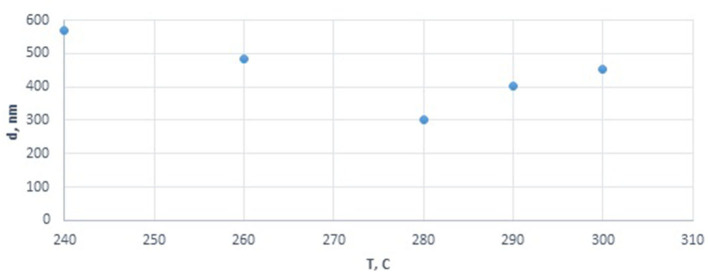
Effect of precursor decomposition temperature on the particle size of cobalt oxide in the cobalt colloids.

We studied the effect of cobalt precursor decomposition conditions on the particle size of the dispersed phase in the final catalyst dispersions at temperatures over that of cobalt nitrate decomposition 208°. This study applied two approaches to generate cobalt-based dispersions *ex situ* (not in the FTS reactor): direct decomposition of cobalt nitrate in the dispersion medium (protocol 1) and slow introduction of an aqueous solution of cobalt nitrate into paraffin pre-heated to the desired temperature (protocol 2). Using the second approach, we evaluated the effect of the pH of the salt solution, which ranged from 4.18 to 4.68. The results are summarized in Table 1 and Figure 1.

**Table 1:** Influence of the conditions of cobalt nitrate decomposition on resulting cobalt-dispersion particle size.

**Precursor**	**Amount of Co, g/100 ml**	**pH**	**Decomposition temperature, °C**	**Average particle size, nm**
Solid Co(NO_3_)_2_·6H_2_O	2	–	215	190
	2	–	230	231
	2	–	245	275
	2	–	260	264
Aqueous solution Co(NO_3_)_2_·6H_2_O	2	4.68	215	584
	4	4.63	215	514
	6	4.53	215	439
	8	4.44	215	333
	12	4.38	215	304
	16	4.18	215	181

**Figure 2:**
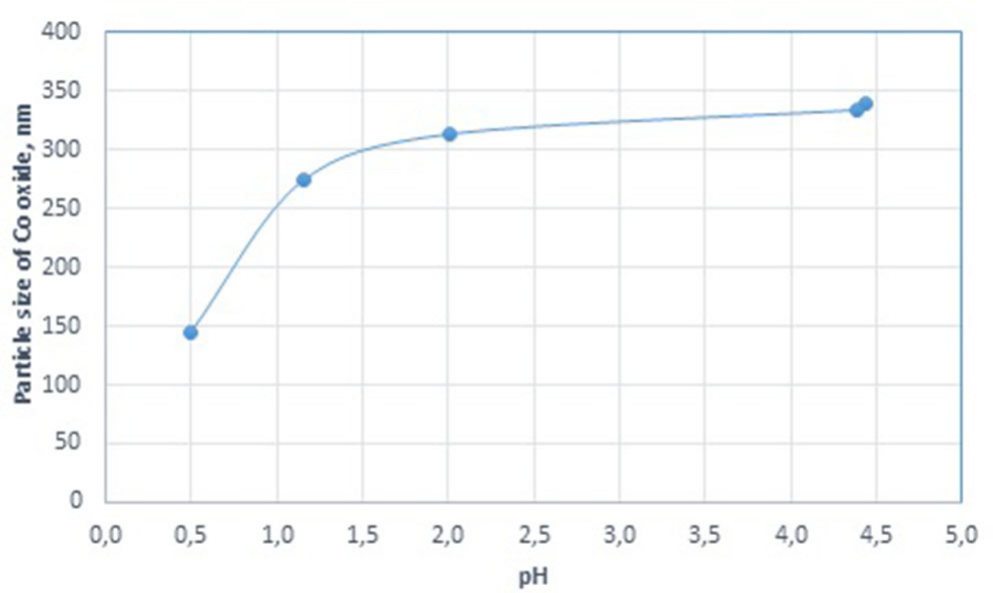
Effect of cobalt concentration on the particle size of cobalt oxide in the cobalt colloids.

Moreover, cobalt-based dispersions formed from highly acidic aqueous solutions of cobalt nitrate introduced into the dispersion medium at 215°C were analyzed. In this case, the acidity of the solutions was adjusted to pH values of 2.01, 1.16, and 0.5 by the addition of concentrated nitric acid. All prepared dispersion systems contained cobalt oxide as the dispersed phase corresponding to 8 g of metallic cobalt. Figure 2 shows the results of this analysis.

Conventional cobalt-based FTS catalysts are generally prepared by the impregnation of aluminum oxide as a support with a cobalt nitrate solution followed by calcination to form cobalt particles deposited on alumina. To investigate the properties of dispersed-phase cobalt-based catalysts with a similar composition, we introduced the cobalt and alumina precursors simultaneously into the molten paraffin. Thus, catalysts with various Co:Al_2_O_3_ weight ratios were synthesized by the decomposition of aqueous solutions of nitrates at 215°C. When preparing the precursor solutions, the concentration of cobalt nitrate was kept constant (10 wt.%), while that of the aluminum component varied (Table 2).

**Table 2:** Effect of aluminum nitrate amount on cobalt-colloid particle size.

**Amount, %**		**pH**	**Average particle size, nm**
**Co nitrate**	**Al nitrate**		
10	30	0.55	344
10	20	0.88	261
10	10	1.34	219
10	5	1.87	345

To study the effect of oxide promoters on the properties of the cobalt-containing dispersions, a series of samples were prepared with aluminum and zirconium oxides at amounts no greater than 30% of the active metal content (protocol 2). The systems were obtained by introducing mixed aqueous solutions of the precursors into the molten paraffin at 280°.

**Figure 3:**
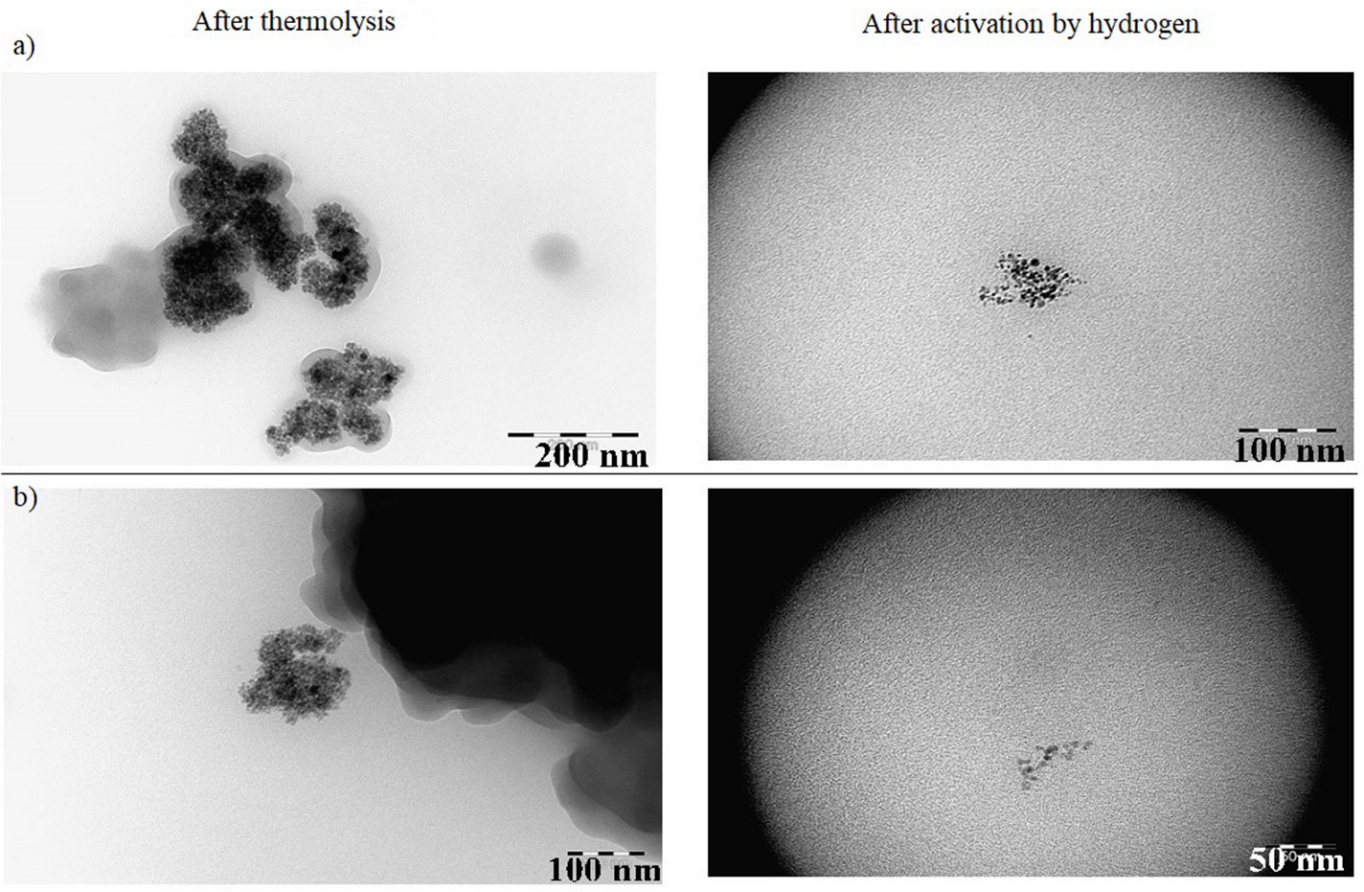
TEM images of samples with **(a)** ZrO_2_ and **(b)** Al_2_O_3_ additives.

Precursor solutions containing 4 g of metallic cobalt were used to study the effect of the amount of ZrO_2_ and Al_2_O_3_ additives on the size of the dispersed-phase particles in the final dispersions. For these systems, the particle sizes were determined after the samples were activated for 5 h under the conditions described in the experimental section (Table 3). To confirm the DLS results, the catalysts were also studied by TEM (Figure 3).

**Table 3:** Dispersed-phase particle size of cobalt colloids with Al_2_O_3_ and ZrO_2_ additives determined by DLS.

**Catalyst**	**Before reduction**		**After reduction H_2_ with 20 atm, 300°C, 20 l/h, 5h**	
	**Size, nm**	**Amount, %**	**Size, nm**	**Amount, %**
100Co:10ZrO_2_	412	100%	9	12%
			475.8	88%
100Co:20ZrO_2_	20	3%	8	53%
	679	97%	232.5	47%
			6	7%
100Co:30Al_2_O_3_	810	100%	588	93%

**Figure 4:**
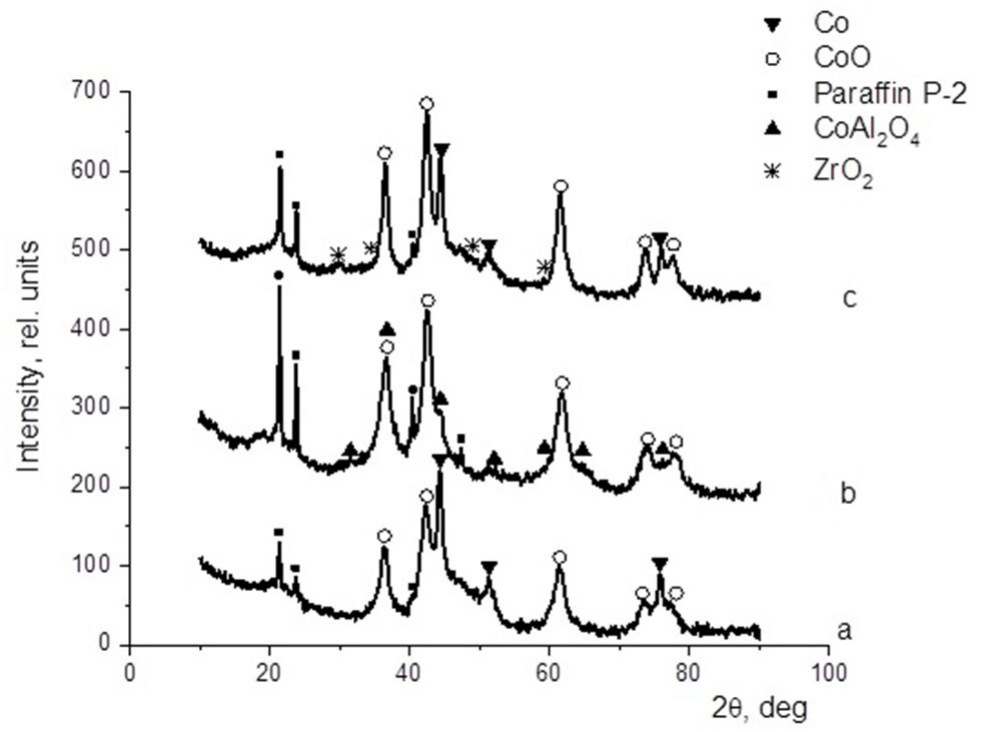
Cobalt colloid surface compositions determined by XRD: **(a)** 100Co, **(b)** 100Co:30Al_2_O_3_, and **(c)** 100Co:20ZrO_2_.

XRD analysis served to characterize the unpromoted and promoted cobalt-based dispersions (protocol 2) after catalysis. In all the XRD patterns illustrated in Figure 4, the presence of P-2 paraffin was evidenced by the peaks at 2θ = 21.5°, 23.8°, 40.3°, and 47.3°. In the pattern of the cobalt-based catalysts (Figure 4a), the characteristic reflections of null-valent cobalt (2θ = 44.2°, 51.5°, and 75.9°) and cobalt oxide (CoO) (2θ = 36.6°, 42.4°, 61.8°, 73.6°, and 77.8°) were found. The introduction of alumina to the catalyst composition at 30% of the cobalt content (Figure 4b) was favorable for the formation of cobalt oxide, and the typical reflections of spinel CoAl_2_O_4_ (2θ = 31.2°, 36.7°, 44.7°, 55.5°, 59.2°, 65.0°, and 77.0°) were observed. On the other hand, the introduction of zirconium (Figure 4c) had almost no effect on the phase composition of the active component, only a slight reduction in the fraction of metallic cobalt. In this XRD pattern, the reflections at 2θ = 29.7°, 34.6°, 49.6°, and 59.2° were attributed to ZrO_2_.

### Rheological Properties of Cobalt-Based Dispersions

**Figure 5:**
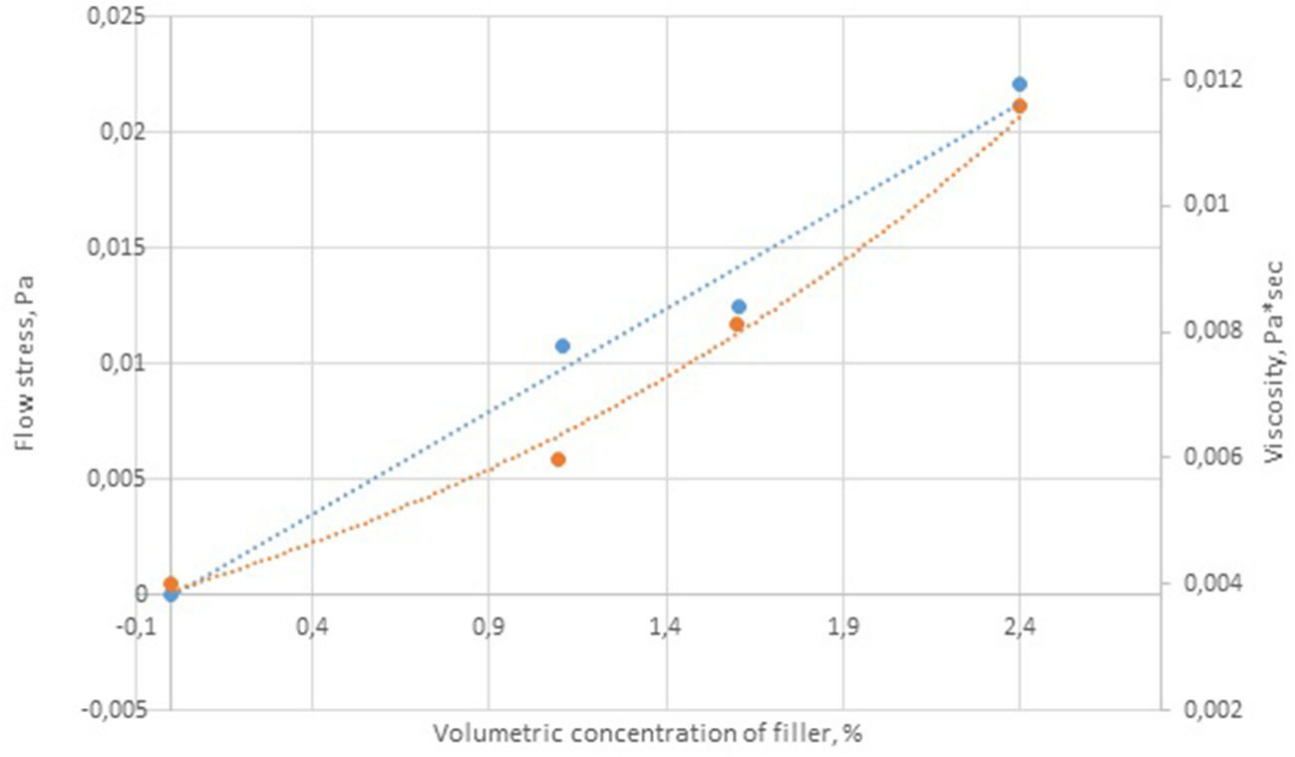
Concentration dependence of viscosity and flow stress of cobalt colloids.

The rheological characteristics of the cobalt-containing dispersions were measured for the samples containing 4–12 g of cobalt. The coefficients of the Bingham equation describing the behavior of the viscosity vs. shear stress curves are given in Table 4. The yield strength correlated linearly with the concentration of the dispersed phase (Figure 5).

**Table 4:** Coefficients of the Bingham equation for cobalt colloids.

**Amount of Co, g**	**τ_0_, Pa**	η^*^, Pa·s
4	0.0105	0.00616
6	0.0116	0.00797
12	0.0233	0.0115

### FTS

The dispersed-phase cobalt-containing catalysts synthesized by injection of the precursor solution directly into the slurry reactor (protocol 3) were activated under H_2_ and then tested for the FTS. Considering that alumina-supported cobalt catalysts are traditionally used for the FTS in fixed-bed or three-phase slurry reactors, in this study, we used alumina as a promoter in the prepared dispersed-phase catalysts. This promoter provided a cobalt-containing catalytic system with additional oxide sites. As a promoter, palladium is known to facilitate cobalt reduction. Thus, the results of the FTS in the presence of dispersed-phase Co, Co–Pd, and Co–Pd–Al_2_O_3_ are summarized in Tables 5, 6.

**Table 5:** Influence of cobalt colloid composition on the FTS (20 atm, 240–320°C, Co:2H_2_, 30 l/h).

**Catalyst (mass%)**	***C*_CO_, %**	***Y*, g/m^3^**	***S*, %**
100Co		Inactive, Xco < 10%	
100Co:2Pd		Inactive, Xco < 10%	
100Co:2Pd:10Al_2_O_3_	44	40	59

**Table 6:** Effect of alumina in ultrafine 100Co:2Pd:5–50Al_2_O_3_ catalyst on the FTS.

**Amount of Al_2_O_3_, mass%**	***T*, °C**	***X*_co_, %**	**Yield, g/m^3^**				***A*, mkmoleCO gCo·s**	***P*, g/(kg_Co_ · h)**	**Selectivity, %**			
			**C_1_**	**C_2_-C_4_**	**C_5+_**	**Co_2_**			**C_1_**	**C_2_-C_4_**	**C_5+_**	**Co_2_**
5	250	12	2	0	6	2	2	28	23	5	68	4
	260	12	6	6	12	3	2	60	24	7	65	4
	270	19	9	2	25	5	3	73	25	8	62	5
	280	30	13	3	36	7	5	178	28	8	59	5
	290	35	23	28	36	12	6	132	32	7	54	7
	300	33	31	13	37	15	7	185	37	7	50	7
15	280	32	14	9	29	6	5	146	26	7	63	4
	290	44	20	12	40	11	6	192	29	7	59	5
	300	41	27	11	36	21	6	150	35	6	52	7
20	250	12	8	0	13	0	2	67	25	1	71	3
	260	22	12	0	27	4	4	136	26	2	70	2
	270	35	16	0	39	4	5	181	28	2	68	2
	280	47	26	4	44	13	8	270	29	2	64	5
	290	49	26	5	42	17	8	225	30	3	60	7
	300	51	38	7	40	33	9	216	37	4	49	10
30	250	5	6	0	7	0	1	14	24	2	70	4
	260	17	13	9	15	3	2	39	26	2	68	4
	270	26	16	11	27	12	3	57	28	2	65	5
	280	39	23	10	39	10	5	97	29	3	61	7
	290	50	38	23	42	24	9	186	30	3	58	9
	300	49	36	8	41	29	9	214	36	4	49	11
40	250	8	3	0	10	2	1	49	25	4	67	4
	260	16	8	1,5	18	3	3	89	26	3	67	4
	270	27	12	8	28	4	9	117	26	7	64	3
	280	38	18	18	36	10	6	181	26	8	61	5
	290	42	23	15	43	13	7	200	29	8	57	6
	300	54	27	13	38	14	9	178	31	6	55	8
40^*^	250	26	9	0	20	5	5	89	15	0	64	2
	260	32	15	2	27	4	4	136	30	2	64	3
	270	36	25	9	33	8	6	163	30	6	60	4
	280	44	31	15	41	14	7	182	30	7	58	5
	290	58	41	11	51	21	10	255	37	4	52	7
	300	57	50	8	40	41	10	179	46	4	42	9
50	250	4	5	0	1	2	1	4	28	1	68	3
	260	13	11	1	14	3	3	72	29	2	66	3
	270	22	12	2	24	4	4	120	32	2	63	3
	280	29	19	3	28	6	5	138	35	3	58	4
	290	37	22	6	29	11	5	132	36	5	53	6
	300	38	28	10	30	16	6	151	39	6	47	8

* *Catalysts prepared using highly dilute solutions*.

**Figure 6:**
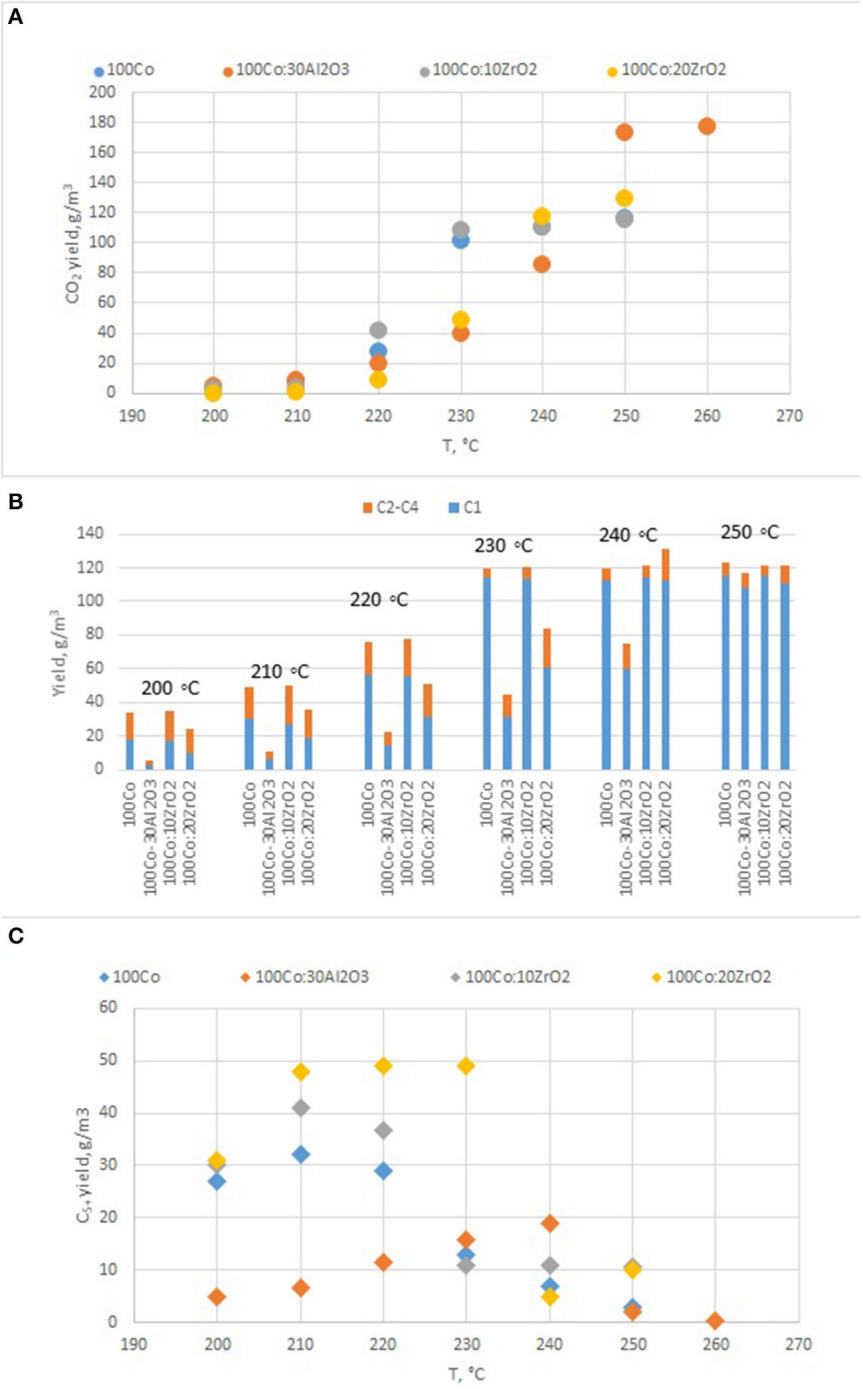
The yield of FTS products in the presence of Co-colloids with Al_2_O_3_ and ZrO_2_ additives. (*P* = 20 atm, CO/H_2_ = 1/2, 20 l/h.). **(A)** CO_2_; **(B)** C_1_–C_5_; **(C)** C_5+_.

**Figure 7:**
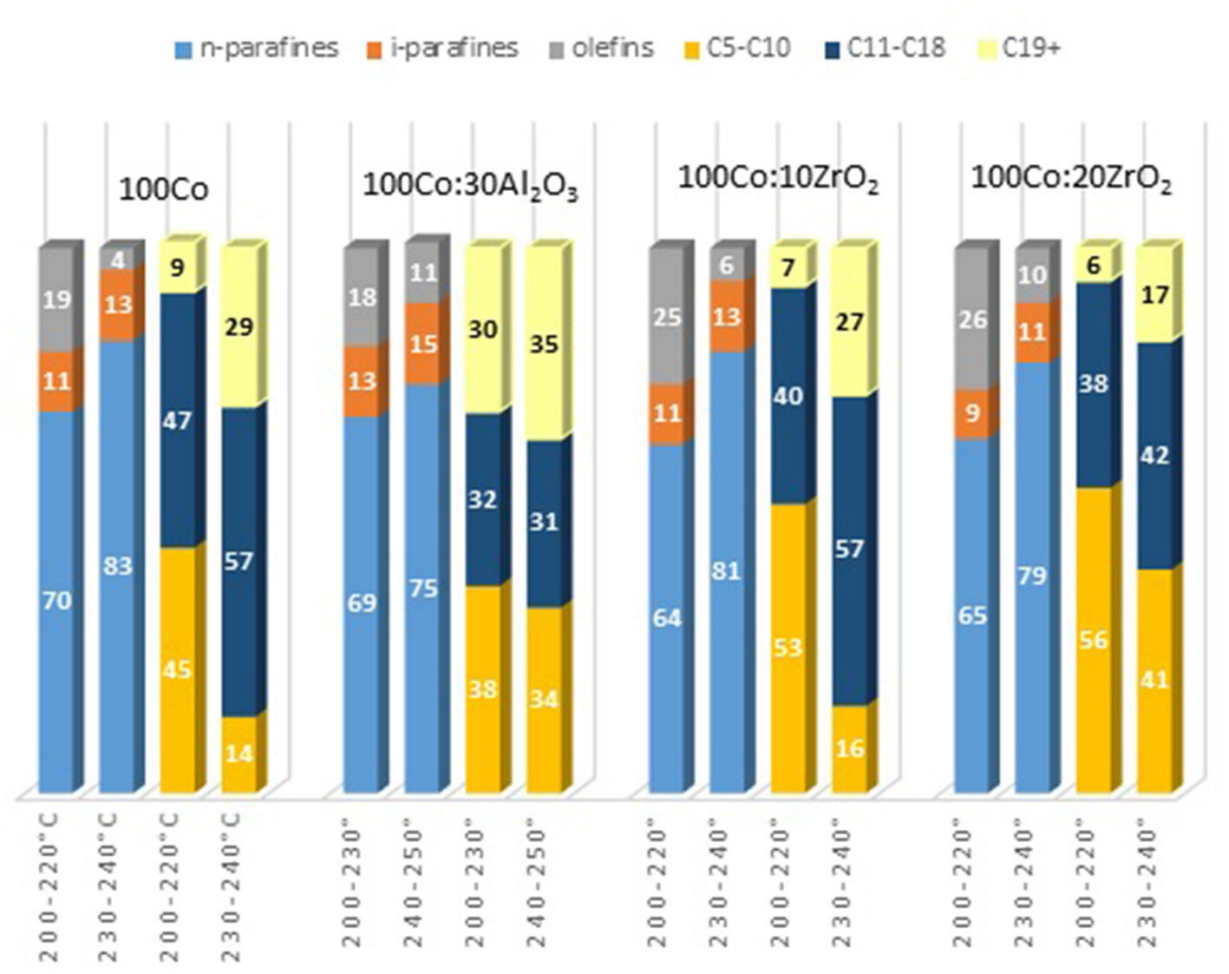
Composition of liquid hydrocarbons obtained by FTS in the presence of cobalt colloids.

To clarify the effect of oxide promoters on the catalytic properties of the cobalt-containing dispersions, a series of samples with Al_2_O_3_ and ZrO_2_ (protocol 2) additives were tested for the FTS. Figures 6A–C demonstrates the yields of the products at various reaction temperatures. The distribution of liquid products obtained from the FTS in the presence of the prepared catalysts is discussed below (Figure 7).

## Discussion

### Effect of Cobalt Nitrate Decomposition Temperature

The decomposition of solid cobalt nitrate in molten paraffin at 215°C resulted in a finely dispersed suspension of cobalt oxide with an average agglomerate size of 190 nm (protocol 1, Table 1). At a decomposition temperature of 245°C, the average particle size of the dispersed phase increased to 275 nm, and above 245°C, a slight decrease in particle size (264 nm) was observed. On the other hand, when an aqueous solution of cobalt nitrate was used to prepare an ultrafine suspension at 215°C, the dispersed-phase particles were significantly larger (up to 584 nm).

Analysis of the effect of the decomposition temperature of cobalt nitrate introduced in the form of a solution with a constant pH of 4.63 (corresponding to 4 g of cobalt in the final dispersion) showed that an increase in temperature from 240 to 280°C decreased the diameter of the dispersed-phase particles from 570 to 300 nm, while a further temperature increase to 300° also increased the size of agglomerate to 450 nm (Figure 1). Thus, the optimum temperature was determined for obtaining the smallest cobalt-containing particles. Deviation from this point seems to lead to agglomeration or sintering of the particles.

#### Effect of Cobalt Content

The effect of cobalt content in the final dispersion (from 2 to 16 g/100 ml) was studied by varying the amount of cobalt precursor in the introduced aqueous solution while keeping the cobalt nitrate solution at a constant concentration (Table 1). It was found that an 8-fold increase in the cobalt content resulted in an almost 3-fold decrease in the dispersed-phase particle size (from 584 to 181 nm).

#### Effect of pH of Cobalt Nitrate Solution

It is generally known that the hydrolysis of cobalt nitrate occurs in aqueous solutions, and the more salt there is in the solution, the lower its pH. When we varied the amount of cobalt in the final dispersion, the pH of the solution introduced into the dispersion medium decreased from 4.68 to 4.18 (Table 1). In this case, a decrease in the size of the final cobalt-based dispersions from 584 to 181 nm was observed. The results shown in Figure 2 demonstrate the effect of a further increase in the acidity of the precursor solution. The smallest particles of dispersed cobalt oxide (144 nm) were formed at a highly acidic pH of 0.5.

### Effect of Alumina and Zirconium Addition to Cobalt-Based Dispersions

Various amounts of added alumina were used to synthesize “model” cobalt-containing dispersions that mimic traditional supported cobalt catalysts (protocol 2, Table 2). Since the degree of hydrolysis of aluminum nitrate is even greater than that of cobalt nitrate, the aqueous solution of cobalt and aluminum salts was more acidic than the solution of cobalt nitrate alone (Table 2). The pH of the mixed solution decreased with the increasing concentration of the aluminum component, and thus the lowest pH (0.55) was observed for the solution with the maximum aluminum content. A minimum was observed in the dispersed-phase particle size of the obtained dispersions arranged in order of increasing pH (from 0.55 to 1.87). The smallest particles (219 nm) were formed in the sample with a weight ratio of Co:Al_2_O_3_ of 1:1.

Previously (Kuz'min et al., 2019; Kulikova et al., 2020), we showed the positive effects of the addition of oxide promoters to Fe-containing dispersions on their catalytic activity. Therefore, in this study, we investigated the impact of small amounts of aluminum and zirconium oxides (≤30% of the cobalt content) on the properties of the dispersed-phase cobalt-containing catalysts. DLS analysis showed that any addition of a modifier increased the particle size of the dispersed phase in the as-prepared cobalt-containing dispersions (Figure 1 and Table 3), although we found that the formation of the dispersed-phase particles of the promoted system ultimately reached completion during treatment under hydrogen flow in the catalyst activation step. The TEM images (Figure 3) showed that in the as-prepared dispersions, the dispersed phase consisted of agglomerates over 200 nm in size, which in turn consisted of smaller grains. During pre-treatment of the dispersion with hydrogen to form catalytically active sites on the dispersed-phase particles [i.e., a mixture of null-valent cobalt and its oxide (Lapidus, 2013), the latter were crushed into grains of <10 nm]. The presence of particles with diameters of 232–588 nm as determined by DLS may be attributable to the magnetic properties of null-valent cobalt causing the agglomeration of small particles.

### Rheological Properties of Cobalt-Based Dispersions

**Figure 8:**
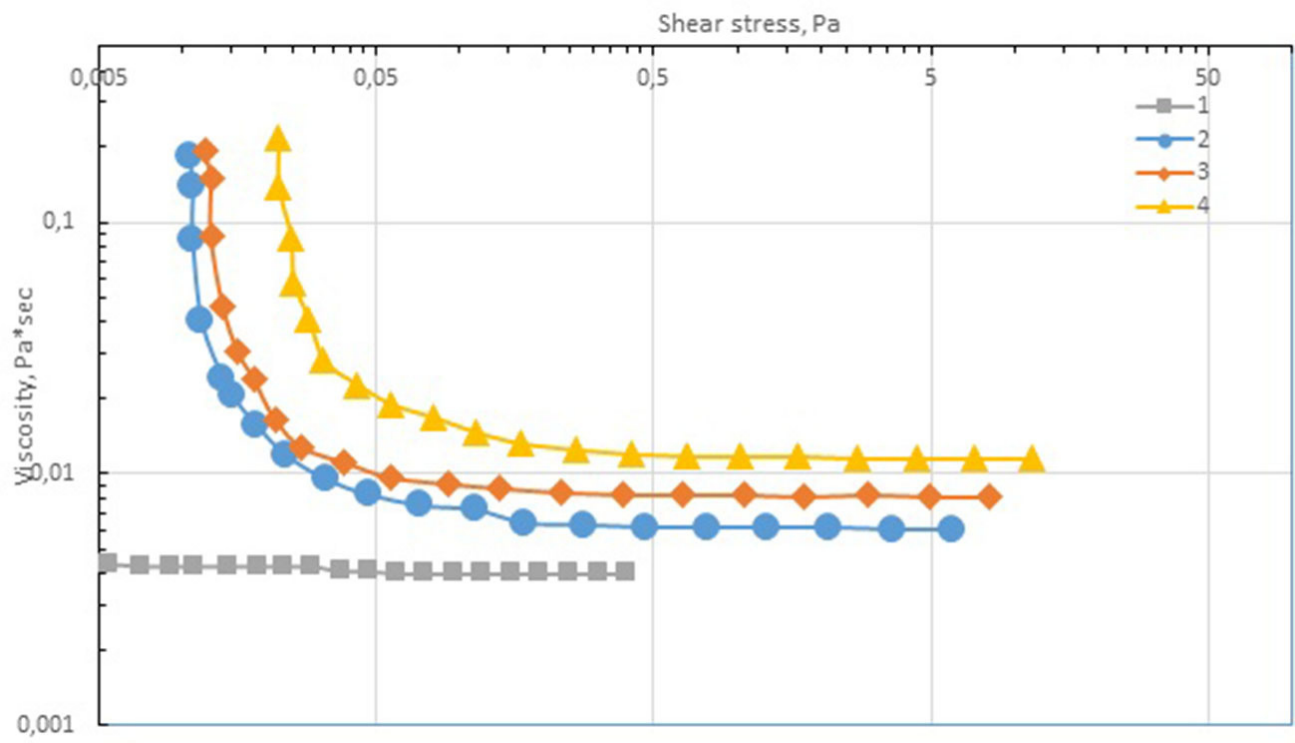
Dependence of viscosity on shear stress. Included cobalt per metal (g): 1–0 (≪pure≫ paraffin); 2–4; 3–6, 4–12.

The rheological analysis of the cobalt-containing suspensions showed that the yield strength was characteristic of all the samples, and it increased (0.011–0.023 Pa) with the content of the dispersed phase (Figure 8). The measured viscosity versus shear stress curves can be described by the Bingham equation:

(1) $$\begin{array}{l} \tau =\tau_{0}+\eta \frac{d\gamma }{dt}, \end{array}$$

where τ_0_ is the yield strength (minimum stress at which a material begins to deform plastically), and η is the plastic viscosity, which characterizes the resistance of the structure to fracture when the stress changes.

Table 4 shows the Bingham coefficients determined for the ultrafine suspensions of cobalt oxide–paraffin. At high shear stresses, the dependence of viscosity on concentration can be represented by the classical power law:

(2) $$\begin{array}{l} \frac{\eta }{\eta_{0}}=1+2.5\phi +x\phi^{2}. \end{array}$$

The second virial coefficient determined for the tested system was quite high (3,280), which indicates a remarkable effect of the dispersed-phase particles on the structure of the dispersion system.

The same dependence can also be represented by the exponential law:

(3) $$\begin{array}{l} \frac{\eta }{\eta_{0}}=a+bexp\left(c\varphi \right), \end{array}$$

where the determined coefficients are as follows: *a* = 0.340, b = 0.645, and *c* = 57.21.

Thus, the cobalt-containing dispersions are viscoplastic materials with a low yield stress, which indicates the formation of a weak structural network in the dispersion medium. The rheological properties of these systems were aptly described by the Bingham equation. Moreover, the studied dispersions are characterized by thixotropic properties (i.e., the samples are destroyed upon deformation, and when repeated stress is applied, the material properties are different from the initial ones).

### FTS With Cobalt-Based Dispersions

The dispersed-phase cobalt-based catalysts prepared *in situ*, [i.e., by injection of the aqueous precursor solution into the FTS reactor (protocol 3), and then reduced for 12 h under hydrogen at 20 atm and 300°C showed no FTS activity in the temperature range of 240–320°C]. The CO conversion did not exceed 10% (Table 5), and no liquid products were obtained. The introduction of palladium into the catalytic system, which promotes cobalt reduction, did not improve the activity of the catalyst. In contrast, the introduction of both alumina and palladium into the catalyst composition afforded an ultrafine cobalt colloidal catalyst with FTS activity (Table 5).

The formation of ultrafine cobalt-based catalysts occurs directly in the dispersion medium by protocol 3 (mix of paraffins). As a result, the cobalt oxide particles are encapsulated (Gubin et al., 2005) which makes it difficult for hydrogen to access the cobalt-containing particles. The addition of small amounts of noble metals (Pt, Ru, and Pd) has a pronounced effect on the properties of cobalt catalysts supported on alumina (Xu et al., 2008) by increasing the degree of contacts reduction at the activation stage and increasing their ability to CO hydrogenation.

The introduction of noble metals into supported cobalt catalysts for Fischer-Tropsch synthesis allows cobalt to stay in a reduced form, preventing its oxidation and deactivation as a consequence. However, the addition of Pd to the Co catalyst did not lead to the expected effect. The CO conversion on this catalytic system did not exceed 10% (Table 5). This effect can be explained by the absence of the cobalt oxide phase in the Co-Pd-containing system.

CO adsorption occurs at the interface between metallic cobalt and the oxide by binding an oxygen atom with the oxide part of the active center (Lapidus and Krylova, 1998). In this case, the C–O-bond strength in the carbon monoxide molecule decreases at the Lewis acid site, which leads to an increase in its hydrogenation rate. Hypotheses about the binary nature of active sites in the synthesis of hydrocarbons from CO and H_2_ were also proposed in works devoted to the study of nickel catalysts (Huang and Schwarz, 1987). As the studies have shown, with the same metal dispersion, the selectivity of the CO hydrogenation process depends on the acidic properties of the support.

This dependence is due to the fact that the growth of the hydrocarbon chain occurs through the interaction of surface CHOH complexes connected via oxygen with a Lewis acid center located on the Co-CoO interface.

CO adsorption studies (Lapidus and Krylova, 1998) show a correlation between the probability of liquid hydrocarbon formation and the presence of weak CO adsorption sites.

The presence of these sites on the cobalt catalysts surface is a necessary condition for the long-chain hydrocarbons formation from CO and H_2_. Systems without acid sites do not catalyze the formation of liquid hydrocarbons due to the absence of surface polymerization centers, which are the cobalt oxide phase. This phase is usually formed during preliminary heat treatment (calcination and / or reduction) of the catalysts as a result of the support (Al_2_O_3_, SiO_2_, SiO_2_-Al_2_O_3_, etc.), cobalt oxide, and the promoter interaction.

In this work, the formation of weak acid sites with an oxide phase was carried out by introducing alumina into the Co-Pd-system.

The addition of 10 wt.% Al_2_O_3_ led to a significant increase in the activity of the catalytic system. CO conversion in this case was 44%. The yield and selectivity of liquid hydrocarbon formation were 40 g/m^3^ and 59%, respectively. Thus, the introduction of modifying oxide additives into dispersed-phase cobalt-based catalysts enhanced the polymerization properties of these systems, and, consequently, increased the selectivity of liquid hydrocarbon formation.

The amount of alumina in the finely dispersed 100Co:2Pd:5–50Al_2_O_3_ catalysts (wt.%) affected the outlet parameters of the FT reaction carried out in the three-phase system (Table 6). At the same time, it should be noted that the alumina content shifted the optimal synthesis temperature (i.e., the temperature at which the largest amount of liquid products was formed). With an increase in the amount of Al_2_O_3_, the optimal temperature increased from 280 to 300°.

It is known (Savost'yanov et al., 2017) that the introduction of alumina into the supported cobalt catalysts affects the contacts catalytic properties, changing the composition of the catalyst active phase and, as a consequence, the selectivity for the resulting products. The effect of the amount of Al_2_O_3_ in the composition of cobalt-based catalysts is presented in Table 6. The increase in the amount of alumina from 5 to 20 wt.% leads to improving the catalytic system activity. In particular, with a 5% Al_2_O_3_ content in the catalyst, the maximum CO conversion corresponded to 35%; with the introduction of 15%, this parameter corresponded to 44%, and with the introduction of 20% alumina, it reached 51%.

A further increase in the content of aluminum oxide in the catalytic system composition up to 40% led to an increase of CO conversion to 54–58%. This tendency may be due to the above-described effect of bifunctional active site formation, which are represented by both the metal and oxide cobalt phases. These areas of the catalytic surface are prone to CO adsorption with the formation of strong bonds. This, according to the authors, can explain the effect of increasing the catalytic activity of dispersed-phase cobalt-based catalysts with 5-40 wt.% aluminum oxide addition. With a further increase in the amount of Al_2_O_3_ to 50 wt. %, the opposite effect from the alumina addition was observed (i.e., reduction of catalytic activity). The CO conversion did not exceed 38%.

A similar effect of the presence of the maximum activity of the catalytic system upon varying the amount of introduced alumina when using a supported contact is described in Pei et al. (2014). This behavior of the catalytic system can be associated with a decrease in the amount of the active cobalt component in relation to the entire catalytically active surface. As a result, the total number of surface areas prone to CO sorption decreases, which leads to a decrease in conversion.

An increment of the total catalytic activity of cobalt-based catalysts with an increase of alumina content in their composition caused an intensification of two main side reactions of the Fischer-Tropsch synthesis—CO_2_ formation and the methanation.

The contact Co-5%Al_2_O_3_ manifested the lowest activity in methane and CO_2_: their yield was 31 and 15 g / m^3^, respectively. These parameters continuously increased simultaneously with the activity of the catalytic system. The introduction of 40 wt.% alumina from dilute solutions resulted in a significant increase in carbon dioxide and methane yield, up to 41 and 50 g/m^3^.

Under the conditions of the Fischer-Tropsch synthesis on dispersed-phase cobalt-based catalysts, as well as on the supported analogs, the main amount of CO_2_ is formed by water gas shift reaction, while the CO disproportionation reaction leading to the deposition of coke on the surface is insignificant (Lapidus et al., 2011).

The intensification of the methanation reaction proceeding in the presence of cobalt colloids promoted with aluminum oxide can be explained by two reasons. On the one hand, the presence of metal centers of weak CO adsorption capacity, which are responsible for the reaction of direct hydrogenation of CO in addition to bifunctional active catalytic sites (Lapidus and Krylova, 1998). On the other hand, an increase in the partial pressure of water due to the intensive course of water gas shift reaction leads to an acceleration of CO hydrogenation and an increase in the yield of methane (Qi et al., 2020). The highest yield of liquid hydrocarbons (45 g/m^3^) was obtained over the catalyst containing 20 parts by weight of aluminum oxide (Table 6), although all the ultrafine Co–Pd–Al_2_O_3_ dispersions were characterized by high productivities of 270–290 g/(kg_Co_ · h). On the other hand, the increase in the synthesis temperature decreased the selectivity of all the catalysts toward the target products (liquid hydrocarbons) due to the noticeable intensification of gas formation in the reaction system, particularly methane. As a result, the selectivity toward C_5+_ products, which was initially ~70%, decreased by ~20%.

The liquid products formed over the cobalt-based catalysts promoted with palladium and alumina were mainly hydrocarbons. The fraction of oxygenates in water did not exceed 5%, and the fraction of paraffins in the hydrocarbon mixtures was over 80%, while the fraction of olefins was less than 10%. The iso-to-normal ratio of the product paraffins was 0.5, which indicates a rather high isomerization activity of the aluminum-containing catalysts. At the same time, the catalysts were also characterized by a high polymerization activity, with an α-value (chain growth probability according to the Anderson–Schulz–Flory model) as high as 0.95. The prepared catalysts enabled the production of hydrocarbon mixtures containing almost 80% waxes from syngas.

As discussed above, the size of the particles in the dispersed phase of the cobalt-containing dispersions depends on the pH and concentration of the aqueous solution of precursor salts. To verify this effect on the catalytic properties of the *in situ* synthesized cobalt-based dispersions promoted with alumina, a 100Co:2Pd:40Al_2_O_3_ catalyst was prepared using a highly dilute precursor solution. The dispersed-phase particles of this dispersion were larger (350 nm) than those of the same dispersion composition prepared from a more concentrated solution (250 nm). However, the dispersed-phase catalyst prepared from a highly dilute solution was more active for the FTS (Table 6).

Analysis of the catalytic properties of the dispersed-phase cobalt-containing catalysts prepared by protocol 2 [i.e., outside the FTS reactor, showed that the introduction of alumina, as a traditional promoter of FTS catalysts, did not enhance the activity of the system; in the temperature range of 200–230°, the CO conversion decreased by a factor of 1.5–5.5 (Figure 6)].

Furthermore, the fraction of gas products increased: the selectivity toward carbon dioxide and light C_2_-C_4_ hydrocarbons increased, whereas the selectivity toward liquid hydrocarbons decreased. However, the selectivity toward methane decreased. In the presence of the catalyst modified with alumina, the highest yield of the C_5+_ fraction was 18.8 g/m^3^, whereas the highest C_5+_ yield was 32 g/m^3^ in the presence of the catalyst without promoters. When FTS was conducted in the presence of the dispersed-phased catalysts modified with zirconium, an improved selectivity toward the target products was observed. The low activity of the system with alumina additives for the formation of liquid hydrocarbons was caused by the absence of null-valent cobalt in the phase composition of the catalyst (Figures 4, 7).

**Figure 9:**
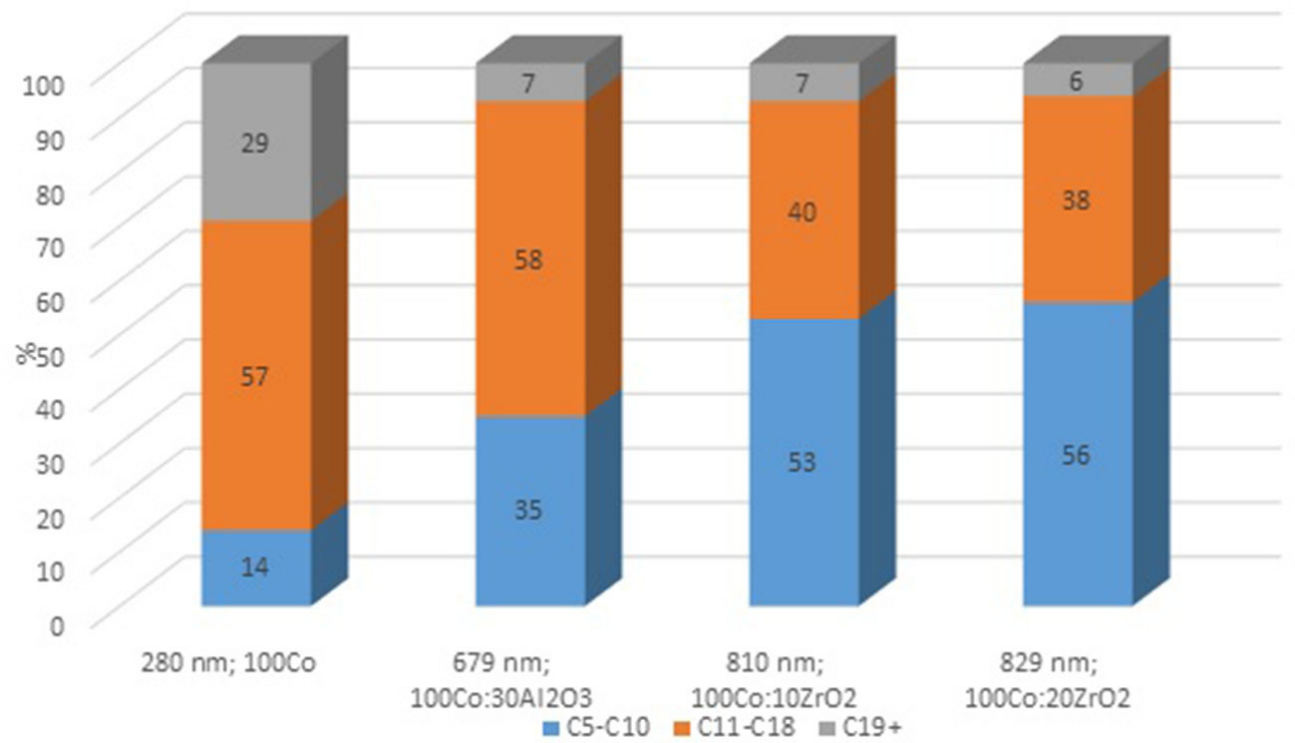
Fractional compositions of FTS products.

The maximum product yield was obtained in the presence of the cobalt-based dispersion with 20 wt.% zirconium oxide additive (Figure 9). The temperature range of 210–230°C seemed to be optimum for the FTS in the presence of the discussed catalysts: within this region, the maximum yield of C_5+_ hydrocarbons was observed, while a further increase in temperature sharply decreased the yield. However, the catalyst with a higher ZrO_2_ content (20 wt.%) demonstrated the maximum FTS catalytic activity. In addition, a correlation was observed between the particle size of the dispersion and the product composition. As the particle size increased, the gasoline fraction increased, and the content of C_11_-C_18_ hydrocarbons proportionally decreased.

## Conclusions

In this work, dispersed-phase catalysts were prepared by the thermal decomposition of solids or aqueous solutions of cobalt salts and promoters introduced into liquid paraffin at temperatures above 215°C, which is the temperature of the decomposition of cobalt nitrate. In this case, dispersion systems containing particles 200–580 nm in size were formed. An increase in the temperature from 215 to 260°C lead to an enlargement of the dispersed-phase particles in size from 190 to 264 nm, which was probably due to enhanced agglomeration.

The ultrafine cobalt-based dispersions prepared from cobalt nitrate aqueous solutions were shown to be viscoplastic materials with low yield stresses, which indicated the formation of a weak structural network in the dispersion medium. Their rheological properties were described by the Bingham equation. Moreover, the systems were thixotropic (i.e., the samples are destroyed upon deformation, and when repeated stress is applied, the material properties are different from the initial ones).

Dispersed-phase cobalt–paraffin catalysts containing palladium were prepared *in situ* in an FTS slurry reactor and were reduced *in situ* under H_2_ at mild conditions (300°C). However, a durational hydrogen treatment was required. This may be because the cobalt–palladium catalysts were hardly reduced; under the operation conditions of the slurry reactor, it could be more difficult to remove water (product of the reduction reactions) from the reaction zone, and the unremoved water may oxidize the formed metallic cobalt. Nevertheless, even in a paraffin medium, the reducibility of the catalysts was ~46%, making this catalyst activation method applicable to the same reactor just before performing the FTS. Ultimately, ultrafine suspensions of 100Co:2Pd:5–50Al_2_O_3_ in paraffin applied as FTS catalysts enabled a yield of C_5+_ hydrocarbons of up to 50 g/m^3^ and productivity of 270–290 g/(kg_Co_ · h).

## Data Availability Statement

All datasets generated for this study are included in the article/supplementary material.

## Author Contributions

AM: writing—review and editing. MK: conceptualization, supervision, and writing—review and editing. OD: formal analysis, draft investigation, and writing—original. AP: investigation, visualization, and data curation. The manuscript was written through contributions of all authors, have given approval to the final version of the manuscript.

## Conflict of Interest

The authors declare that the research was conducted in the absence of any commercial or financial relationships that could be construed as a potential conflict of interest.
